# Young pregnant women's views on the acceptability of screening for chlamydia as part of routine antenatal care

**DOI:** 10.1186/1471-2458-10-505

**Published:** 2010-08-19

**Authors:** Jade E Bilardi, Deborah L De Guingand, Meredith J Temple-Smith, Suzanne Garland, Christopher K Fairley, Sonia Grover, Euan Wallace, Jane S Hocking, Sepehr Tabrizi, Marie Pirotta, Marcus Y Chen

**Affiliations:** 1 Sexual Health Unit, Melbourne School of Population Health, The University of Melbourne, Carlton, Victoria 3053, Australia; 2 Melbourne Sexual Health Centre, Alfred Health, Carlton, Victoria 3053, Australia; 3 Primary Care Research Unit, Department of General Practice, The University of Melbourne, Carlton, Victoria 3053, Australia; 4 Department of Clinical Microbiology and Infectious Diseases, Royal Women's Hospital, Melbourne, Australia; 5 Department of Obstetrics and Gynaecology, The University of Melbourne, Melbourne, Australia; 6 Department of Obstetrics and Gynaecology, Monash University Faculty of Medicine, Nursing and Health Sciences, Monash Medical Centre, Melbourne Australia; 7 Centre for Women's Health, Gender and Society, Melbourne School of Population Health, The University of Melbourne, Melbourne, Australia

## Abstract

**Background:**

In pregnancy, untreated chlamydia infection has been associated with adverse outcomes for both mother and infant. Like most women, pregnant women infected with chlamydia do not report genital symptoms, and are therefore unlikely to be aware of their infection. The aim of this study was to determine the acceptability of screening pregnant women aged 16-25 years for chlamydia as part of routine antenatal care.

**Methods:**

As part of a larger prospective, cross-sectional study of pregnant women aged 16-25 years attending antenatal services across Melbourne, Australia, 100 women were invited to participate in a face-to-face, semi structured interview on the acceptability of screening for chlamydia during pregnancy. Women infected with chlamydia were oversampled (n = 31).

**Results:**

Women had low levels of awareness of chlamydia before the test, retained relatively little knowledge after the test and commonly had misconceptions around chlamydia transmission, testing and sequelae. Women indicated a high level of acceptance and support for chlamydia screening, expressing their willingness to undertake whatever care was necessary to ensure the health of their baby. There was a strong preference for urine testing over other methods of specimen collection. Women questioned why testing was not already conducted alongside other antenatal STI screening tests, particularly in view of the risks chlamydia poses to the baby. Women who tested positive for chlamydia had mixed reactions, however, most felt relief and gratitude at having had chlamydia detected and reported high levels of partner support.

**Conclusions:**

Chlamydia screening as part of routine antenatal care was considered highly acceptable among young pregnant women who recognized the benefits of screening and strongly supported its implementation as part of routine antenatal care. The acceptability of screening is important to the uptake of chlamydia screening in future antenatal screening strategies.

## Background

Genital *Chlamydia trachomatis *infection is one of the most common bacterial sexually transmitted infection (STI) worldwide [1]. Untreated chlamydia infection can cause serious reproductive sequelae for women, including pelvic inflammatory disease, tubal infertility, chronic pelvic pain, and ectopic pregnancy [2,3].

In pregnancy, untreated chlamydia infection has been associated with adverse maternal and neonatal outcomes, including increased risk of miscarriage, postpartum endometritis, premature rupture of the membranes and preterm labour for the mother, and a risk of low birth weight and transmission during the delivery to the neonate, leading to conjunctivitis and pneumonitis [4-8]. Most women infected with chlamydia do not report symptoms, and are therefore unlikely to be aware of their infection [3].

Cross-sectional studies in developed countries have shown chlamydia prevalence can be high among pregnant women, particularly in teenagers and young women under 20 years of age [9,10]. In a UK study by Norman et al (2004) the prevalence of chlamydia among pregnant women under the age of 20 years attending antenatal clinics was 12.1% [11]. In a recent Australian study by Chen et al (2009), the prevalence of chlamydia in a cross section of pregnant women aged 16 to 20 years attending antenatal services across Melbourne was 6.4% [12].

The US Preventative Services Task Force has found evidence of improved pregnancy and birth outcomes for pregnant women who are treated for chlamydia and recommend screening of all pregnant women aged 24 years and younger [13]. A number of countries recommend the screening of pregnant women aged 25 and less, however the extent to which this screening is undertaken varies within and between countries [14-16]. There are however, no randomized controlled trials on the benefits and outcomes of screening for chlamydia during pregnancy. Currently in Australia, pregnant women are generally not screened for chlamydia as part of their routine antenatal care although selective screening of teenage girls occurs at some services.

Few published data are available on whether young pregnant women would find screening for chlamydia an acceptable part of routine antenatal care [17,18]. The acceptability of chlamydia screening during pregnancy would be critical in the success of programs aimed at improving screening of this population of women. The aim of this study was to determine the acceptability of screening young pregnant women aged 16-25 years for chlamydia as part of their routine antenatal care.

## Methods

Women were recruited from four major antenatal services across Melbourne (population 3.9 million) [19] Victoria, Australia between December 2006 and July 2007: the Royal Women's Hospital, Mercy Hospital for Women, Sunshine Hospital and Southern Health. Women were recruited as part of a larger cross sectional study examining the prevalence and factors associated with chlamydia in pregnancy [12]. As part of the larger cross sectional study, women were recruited from a broad range of areas across Melbourne to capture the socio-economic, cultural and geographical diversity of the population. In this study, any women aged 16-25 attending services were eligible for the study. At the time of recruitment, women were provided information and informed by a research nurse about chlamydia and the associated adverse maternal and neonatal outcomes of untreated chlamydia infection. Women were asked to provide a first void urine specimen for chlamydia testing by polymerase chain reaction and complete a brief questionnaire providing basic demographic information, recent sexual behaviour, genital symptoms and recent antibiotic use. At the time of recruitment, routine antenatal screening for chlamydia was not offered at any of the participating services.

At the time of recruitment, women were informed and provided consent to be approached at a later date for an interview on the acceptability of screening. Women were approached for the acceptability interview after they had been tested for chlamydia, received their test results, where applicable, been treated for chlamydia with single-dose oral azithromycin (1.0 g) and had sufficient time to notify their partner/s.

Women returning for an appointment at all four services were approached opportunistically to participate in the acceptability interview following their scheduled appointment. Women's disease status was known in advance to allow researchers to approach all chlamydia infected women. To participate in the sub-study women were required to have a reasonable command of English.

Those who consented were interviewed in a private room within the service. People accompanying the participants were asked to remain in the waiting room while the interview was conducted. Participants were offered two cinema tickets for their participation in the interview.

All interviews were conducted face-to-face by JB using a semi-structured interview schedule that contained 21 specific questions, most of which were open ended and allowed the interviewer to probe for further clarification or questioning. The interview schedule consisted of primarily open ended questions about participants knowledge of chlamydia, self-assessed on entry to the larger study and again at the time of the acceptability interview, their experience and opinion on antenatal screening, specimen collection and receiving results, the experience of testing and treatment (if applicable) and informing partners (Additional file 1). Questions were also slightly modified depending on whether the participant had received a positive or negative test result. On average, interviews took approximately 10 to 15 minutes to complete.

All interviews were digitally recorded, transcribed verbatim and imported into N-Vivo 8 for thematic analysis. All data were de-identified to protect confidentiality. JB and DD reviewed the transcripts separately before meeting to discuss codes and emergent and recurrent themes. A coding system was derived from the interview schedule questions and themes arising recurrently in the interviews. Similar codes and initial themes were grouped into broader topics i.e. knowledge of chlamydia, acceptability of screening, and examined for similarities and differences. Ten percent of transcripts were further reviewed independently by MTS to confirm coding and themes. Demographic data were partially obtained from initial questionnaires completed by participants as part of the larger study. Analyses of demographic data were conducted using SPSS 17.0.

Ethical approval for the study was granted from Research Ethics Committee at each of the four hospitals and The University of Melbourne.

## Results

A total of 101 women were invited to take part in the study, of which 100 participated (99% participation rate). One 24 year old woman who had tested positive for chlamydia declined to participate. Of the 100 women who participated, 69 had tested negative for chlamydia and 31 positive for chlamydia. The characteristics of participants are shown in Table 1.

**Table 1:** Characteristics of pregnant females aged 16-25 years (n = 100)

*Participant characteristics*	% or Median [range]
Age	22 [16-25]
Weeks gestation	22 [6-42]
Place of birth	
Australia	69
Outside Australia	31
Aboriginal or Torres Straight Islander	3
Education level	
Year 9	13
Year 10-12	55
Tertiary	32
Relationship status	
Married	42
Living with partner	37
Not living with partner	16
No partner	5
Tested previously for chlamydia	9
Chlamydia test result	
Negative	69
Positive	31
New sex partners in last 12 months	
Yes	13
No	87
Number of sex partners last 12 months	
1 partner	80
2-3 partners	18
4+ partners	2

Women who tested positive for chlamydia were more likely to be under 20 years of age (32% versus 15%, p = 0.04), have had a new sexual partner in the last 12 months (26% versus 7%, p = 0.01), had two or more partners in the last 12 months (44% versus 9%, p < 0.01) and be unmarried or single (77% versus 49%; p = 0.01).

### Women's knowledge of chlamydia

In general, women had low levels of awareness of chlamydia prior to the study. Two thirds of women had never heard of chlamydia before or only knew of it by name, with one third aware it was an STI but unable to provide any further details. Only five women knew it was an STI and were able to provide a small amount of detail about the infection:

*...I didn't know anything about it and then when [the research assistant] told me to read about it, and I read it, and then when I found out I was positive it just shocked me [because] I didn't even know I could have it*. (*KS387*)

...I only knew that it was an STD, I didn't know what it was, or any specifics about symptoms or anything like that. (JH241)

Women's main sources of information were school sex education, friends, women's magazines or through previous testing. Misconceptions around chlamydia transmission, testing and sequelae were common, and included beliefs that: testing was routinely undertaken concurrently with pap smears; transmission could occur from towels, toilet seats or through non-sexual means; chlamydia infection was 'like herpes'; men are the 'carriers'; and that it is an untreatable infection.

...Actually I think you can catch chlamydia non-sexually can't you? (SA222)

...What I heard [about chlamydia previously] was completely different to what I actually found out about [it]...I just thought it would show up in a pap smear. (ME465)

A number of women associated risk of infection with high numbers of sexual partners or sexual promiscuity, believing that women with only one or two lifetime sexual partners were not at risk of infection.

Of note, women retained little knowledge even after being informed and tested for chlamydia. Women, who had been tested previously for chlamydia, or tested positive as part of the study, were more likely to retain information.

### Acceptability of screening

All women, regardless of their relationship status, duration of pregnancy or whether they had been tested previously for chlamydia, supported testing for chlamydia as part of their routine antenatal care. Overall, women felt that being tested for chlamydia was 'no big deal', particularly given the number of routine tests and checks undertaken as part of their antenatal care and supported screening for all pregnant women regardless of age.

*...Well you [get] tested for everything else, all these other things that can harm the baby, don't you? I mean there's a test for Hep B, HIV, all these other things that can harm the baby. I mean if chlamydia is an infection that can harm the baby, go for it. Why would you want to put your baby through any [potential harm]? *(MB304)

...I'm not just saying it but I'd recommend it. I can't see any downfalls to it...you can if you don't have it [the test], there's a lot of downfalls, but "Have a test", it's no biggie. (CK108)

...I'd want to know about it definitely, so that I didn't pass it on and also so that I could get treated for it, especially if you don't know it is there... (PA196)

Nearly all women reported that they strongly preferred urine testing over any other forms of specimen collection such as vaginal or cervical swabs because it was quick, easy and non-invasive.

...it's just simple and easy and the other ways are a bit uncomfortable. (LD203)

...It's convenient, it's not too invasive, it's something you can go and do privately, it's pretty routine. (JH241)

Almost all women bar one reported that they would recommend chlamydia testing to a friend who was pregnant.

...I was actually talking to my sister-in-law about that chlamydia today, this morning, because she's pregnant too and she's definitely going to get that [test] done too. (AG244)

...I've actually already recommended it, like to my younger cousins who are more sexually active than me, and they are not pregnant. Because it's something you do, if they don't do it now they are not going to be able to fall pregnant, there is that chance. (MS465)

### Motivation for supporting screening

The main motivating factor in the acceptability of screening was concern for the health of the baby. The safety of the baby was paramount, with women reporting they would undertake whatever care was necessary to ensure their baby was born without any health problems or complications. Women felt the benefits of screening for themselves and their babies far outweighed any potential concerns about testing.

...Oh anything for the baby, as long as my baby's healthy I will do anything for my baby. I wouldn't risk my baby having an infection. (RA926)

Women's main concern about testing for chlamydia was whether testing and treatment could potentially harm the baby. A few women reported that they were a little embarrassed about being asked to take the test or a little nervous or apprehensive about the treatment, however once assured that testing and treatment was safe during pregnancy, all women were happy to undertake testing.

...You want to get it done [test] to see if you have chlamydia but you think, 'My God, what about the baby?', so you don't want to do any harm to your baby either. (AM243)

...That was my main concern, that I was safe to be treated while I was pregnant without risking anything happening to the baby, and as long as that was fine I was willing to be treated while I was pregnant...just as long as it didn't harm the baby. (TA1061)

Women commonly questioned why screening was not already being done alongside other routine antenatal STI screening given the adverse outcomes for both mother and baby.

### Trust in their physician

Women tended to implicitly trust their physician to know what was best for them, and their baby, stating that they would undertake whatever care or testing was recommended by their physician during pregnancy. While it could be uncomfortable or embarrassing at first, women reported that they would not mind their physician asking for a sexual history to assess their risk of chlamydia mainly because they were pregnant and therefore obviously sexually active, the request would be for pregnancy related medical reasons, the information would be confidential and because they were generally in committed, monogamous relationships.

...It's fine for me because if the doctor thinks he or she needs that kind of information to enable them [to] make any recommendation then it is OK to ask this question. (CW249)

A few women noted that if they were single, had their partner in the room or felt that they were being judged by their physician they would not be comfortable in providing a sexual history.

...Um...oh twelve months ago I probably wouldn't have liked being asked that question but considering I've been with my partner and only him for the last 12 months, it doesn't bother me. It could be a bit intrusive and you could feel like it could be a bit demeaning cos people tend to judge you if you've had a few. (SS335)

### Chlamydia infected women

Chlamydia infected women did not noticeably differ from uninfected women in their responses and attitude toward screening, other than voicing noticeably stronger support for screening as a result of their diagnosis and being more likely to retain information about chlamydia than uninfected women. Women who tested positive for chlamydia had mixed reactions; however, most felt relief and gratitude at having had chlamydia detected. While some women reported feeling shocked at first, very few experienced strong, negative reactions around the diagnosis.

They took a pragmatic approach to the diagnosis, gathering information about chlamydia and resolving to get treatment quickly for the health of themselves and the baby. The majority of women expressed relief and gratitude at having been diagnosed and treated.

...I am really happy that I went in the study cos otherwise I wouldn't have a clue. I'm really glad that I found out now and got treatment and stuff and hopefully it's gone. (TM1061)

...I'm just glad that, you know, I seen you, so that I found out that I had [the] infection and it's going to be treated now, so thank you. (KK529)

Most women sought treatment quickly and reported no adverse side effects to the treatment for chlamydia (single-dose oral azithromycin 1.0 g). Of the few women who reported side effects, the most common side effects included nausea, vomiting or stomach cramps. Overall, women's main concern around treatment was again the safety of the baby.

...that was my main concern, that it was safe to be treated while I was pregnant without risking anything happening to the baby and as long as that was fine I was willing to be treated while I was pregnant... (TM 1061)

The exception was one woman who was very complacent about treatment, reporting that she had not yet been treated for chlamydia as it would be an inconvenience and she did not feel she was infected.

...I really don't know [if I will get treated], it's really inconvenient for me because I have a little baby and there if nobody to take care of [him/her].... I am not worried at all. I don't feel I have an infection. Yeah I do [believe the test] but I'm just not worried about it. (NA701)

Two women reported that their partners had not sought treatment or it was unlikely they had been treated despite being informed of their risk of chlamydia.

### Partner notification and support

The majority of women informed their partners of their chlamydia diagnosis and reported high levels of partner support. In general, women were comfortable informing their partner of their diagnosis because they were in committed relationships, felt they had a moral obligation to stop re-infection and transmission to others and were confident that their partner would be supportive.

...I discussed it with him once I found out that I was infected...um, because I thought he had a right to know because he would have been infected as well and we both went off and got the antibiotics and stuff like that... (TM 1061)

...it was easy for me because I've been with my partner for a while and, you know, we knew everything was alright and, you know, we could have had this chlamydia before we even met, so we had no problems. He went to a GP that night after I found out and got tablets straight away as well, so I had no problems, we had no problems with each other. (AS354)

Overall, women reported that neither themselves, nor their partners reacted angrily to the diagnosis or blamed one another; however, a couple of women did question their partners following the diagnosis.

...He was pretty fine with it, he just had to get treatment straightaway. I did question [him on] a lot of stuff but it was OK and then we had this honesty thing, how he had to tell me everything...yeah it turned out good. (KS387)

...I actually rang him and told him that, well, this is the case, I've got it, questioned him a little bit. But at the end of the day we said 'Well, I need to get my antibiotics, you need to go to the doctors, and it took him a while, and I said "Well, there's no sex in between" and so he went to the doctors. So once I told him he was pretty much as shocked as I was and um, yeah, we both sort of discussed it, it was easy to fix and yeah, we were lucky we did get it picked up when we did. (MS465)

A few women did experience some negativity around notifying their partners, mainly in terms of their partners showing little care or interest in the diagnosis or not believing them at all.

*...Well I got in contact with him [and] he didn't really seem too "Oh God, you know, that's bad", it's just, like he didn't really care, said it's not his problem in other words, that's how he made it sound. I told him that you know, you can go and get it checked up, I spelt it [out] to him over the phone so that he could go to the doctor, and he said "Nah, you know, what for?" He said, "I'm alright"*.* (MH262)*

Overall, at the time of the interview, three women, two single and one in a relationship, reported that they had not informed their partner about their risk of chlamydia, either because the relationship had broken down, their partner was overseas or because of infidelity on their part.

## Discussion

In this study we found that chlamydia screening, as part of routine antenatal care, was considered highly acceptable among young pregnant women, who were strongly motivated by concern for the health of their baby and willing to undertake whatever care was necessary to ensure the safety of their baby. Women commonly questioned why chlamydia testing was not already being undertaken as part of routine, antenatal care given the possible complications of untreated infection for mother and baby. Urine testing was preferred as is it was quick, easy and non-invasive. As previous studies have shown [9,10,12,20,21], chlamydial infection was associated with a young age, more than one recent sexual partner and unmarried status. Women who tested positive for chlamydia were relieved and grateful to have been diagnosed and receive treatment and reported high levels of partner support.

### Strengths and limitations

There are few data on the acceptability of chlamydia screening among pregnant women. To our knowledge, this is the first study to specifically explore the acceptability of screening for chlamydia after test results are received among young, pregnant women as part of routine antenatal care. Previous studies have focused on testing method preferences of pregnant women [22,23] and the acceptability of screening among pregnant teenagers using self-administered vaginal swabs [17]. Strengths of this study include the number of women interviewed, inclusion of chlamydia infected women and the high participation rate (99%).

A limitation of the study was the depth of data collected. Traditionally, conventional qualitative interviews take between 30 to 60 minutes, with schedules guided by broad research topic/s, allowing for open, flexible in-depth discussion of the topic/s. While our questions were predominantly open-ended, we chose to use an interview schedule of 21 fixed questions to ensure we collected specific information relevant to our topic. The fixed nature of the interview schedule and interviewing women 'on the spot', meant interviews were relatively short and lacked the depth of conventional qualitative interviews as women were limited for time; however, this also resulted in a high participation rate.

Further limitations of our study were that women were only recruited from sites in Melbourne, Australia, were not randomly selected, and were all in the 16-25 year age group, therefore their views and experiences of antenatal screening for chlamydia may not be representative of pregnant women elsewhere.

Past research has shown that women are more likely to find chlamydia screening acceptable if they are well informed of the serious sequelae of untreated chlamydia, understand that it is a common condition which is often asymptomatic, and are aware it can be easily treated [24]. A personal factor also found to facilitate women's acceptance of screening is a belief they may be pregnant [24]. Common barriers to chlamydia testing include: denial by women of sexual activity or risk of infection; a reluctance to be tested because of moral connotations and stigma associated with a positive diagnosis, feelings of shame, guilt, embarrassment and anger; a fear and anxiety around future reproductive health and the effects of a positive diagnosis on partner relationships; concerns around privacy and confidentiality; and the time, cost and method of sample collection [24].

It is likely that chlamydia testing was highly acceptable to pregnant women in this study because the test was simple, safe and non-invasive [10,22,23]; one of a number of screening tests undertaken during pregnancy and women's concern for the health of their baby was paramount. Interestingly, the same levels of shame, guilt, fear, anger and embarrassment reported by non-pregnant women following a positive diagnosis and the notification of partners were generally not experienced by women in this study [24-29]. While reactions were mixed, most women took a very pragmatic approach to their diagnosis, treatment and the notification of partners. Negative reactions to the diagnosis and partner notification were experienced by few women, with most reporting high levels of partner support. This is possibly because most women were sexually active within a committed long term relationship which provided them with a sense of security, support, high levels of trust and non-judgment from both partners and health professionals. Women in this study certainly seemed to feel less pressure around the moral connotations and stigma commonly associated with a positive diagnosis. Interestingly, the few single women in the study reported similar views and experiences around testing and treatment, suggesting that the health of the baby also took precedence over any stigma related concerns single women may have had.

While screening for chlamydia was highly acceptable among women in this study, if screening is to be broadly accepted and implemented as part of antenatal care, further education and awareness is required. Women in this and previous studies [17,30] have commonly shown low levels of awareness about chlamydia. Accurate information and education plays a major role in the acceptability of testing for chlamydia, with women more likely to accept testing if they know chlamydia is a serious, common infection that is often asymptomatic and can cause significant reproductive sequelae [24].

## Conclusions

Chlamydia testing using urine specimens was highly acceptable among young pregnant women in this study. For antenatal chlamydia testing to be broadly accepted it is imperative pregnant women are well informed about chlamydia and the benefits of screening. The acceptability of testing is vital in the successful uptake of screening and informing appropriate antenatal screening strategies.

## Competing interests

This study was funded by the Australian Commonwealth Department of Health and Ageing through a Chlamydia Pilot Program Targeted Grant. MC and JH were supported by NHMRC fellowships 400399 and 359276, respectively.

## Authors' contributions

JB was responsible for patient recruitment, data collection and analysis and preparing the first draft of the manuscript. DDG was responsible for patient recruitment, data analysis and critical review of the manuscript. MTS contributed to the design and planning of this study, assisted with the qualitative data analysis and critically reviewed the manuscript. SG contributed to the conception, design and planning of this study and critically reviewed the manuscript. CF contributed to the conception, design and planning of this study and critically reviewed the manuscript. SG contributed to the conception, design and planning of this study and critically reviewed the manuscript. EW contributed to the conception, design and planning of this study and critically reviewed the manuscript. JSH contributed to the conception, design and planning of this study, assisted in the statistical analysis and critically reviewed the manuscript. ST contributed to the conception, design and planning of this study and critically reviewed the manuscript. MP contributed to the conception, design and planning of this study and critically reviewed the manuscript. MYC contributed to the conception, design and planning of this study and contributed to the writing, editing and approval of the manuscript. All authors read and approved the final manuscript.

## Pre-publication history

The pre-publication history for this paper can be accessed here:

http://www.biomedcentral.com/1471-2458/10/505/prepub

## Supplementary Material

Additional file 1 **Interview schedule**. Schedule used to interview women participating in the study. Click here for file
