# Supplementary material for: Young pregnant women's views on the acceptability of screening for chlamydia as part of routine antenatal care
Source: BMC Public Health. 2010 Aug 19;10:505. doi: 10.1186/1471-2458-10-505 (PMC2933724; doi:10.1186/1471-2458-10-505)
Supplement: Additional file 1 — Interview schedule. Schedule used to interview women participating in the study. [file 1471-2458-10-505-S1.DOC]

**STUDY CODE 🖵🖵🖵🖵**

**ACCEPTABILITY QUESTIONNAIRE**

## Preamble

## Thank you for taking part in this important study. We are aiming to find out whether pregnant women in Australia should be offered testing for chlamydia and how they feel about this. We would like to ask you some questions about this.

This questionnaire is anonymous – we will not record any details that can identify you.

## Questions

PRIOR TO SCREENING

**1. What did you know about “chlamydia” before you were asked to take part in this study?**

Prompt: If say they knew nothing can ask **‘Had you heard of it though’?**

🖵 Yes 🖵 No

POST SCREENING

**2. What do you now know about chlamydia?**

[The researcher will explore the following points:]

- Transmission
- Symptoms or warning signs
- Long term physical problems
- Treatment
- Incubation period

EXPERIENCE OF SCREENING

**3. Do you see any benefits in screening for Chlamydia in pregnancy?**

**4. How did you feel about having a chlamydia test as part of your antenatal care?**

SELECTIVE SCREENING

**5. It is recommended that all women under the age of 25 who are sexually active should be screened for Chlamydia. In your opinion, should screening be offered**

(a) To *all* pregnant women aged 25 and under OR

(b) To pregnant women aged 25 and under *who have had more than one recent sexual partner*

[If the woman asks what “recent” means, say “in the last 12 months”]

**Why?**

**6. Do you think pregnant women over the age of 25 should be offered a chlamydia test?**

🖵 Yes 🖵 No

**Why?**

RECOMMENDATIONS ON SELECTIVE SCREENING

**7. In order for the doctor looking after your pregnancy to decide whether to recommend screening they may ask you how many sexual partners you have had in the last 12 months?**

**How would you feel about this?**

**8. There are different ways chlamydia can be tested for:**

1) A urine test (like the one you provided)

2) A cotton bud swab that is inserted into the vagina by the doctor

3) A cotton bud swab that you insert into the vagina

4) A tampon that you insert into the vagina

**Given you are pregnant, which method of testing would you have preferred** (circle response above)?

[Probe: Easier? More acceptable? Any concern about harming the unborn child?]

**Why?**

**9. If a female friend asked your advice, would you recommend that she have a chlamydia test when she is pregnant?**

🖵 Yes 🖵 No

INFORMATION PROVISON AROUND SCREENING

**10. What do you think pregnant women should be informed of before they are tested for chlamydia?**

[Probe: Pre-test counselling? Other particular information to be provided?]

**11. How would you have preferred to receive your test result?**

[Probe: By whom?]

PARTNER Q’s

**12. Did you discuss having your chlamydia test with your partner?**

🖵 No – Don’t have a current partner

🖵 Yes told partner – Go to Q.13

🖵 No didn’t tell partner – Go to Q.14

**13. Could you tell us about your experience in telling your partner about the test?**

**Q. 14 If you did not tell your partner, please tell us why.**

RESULTS

*[The following questions vary depending on whether the participant had* ***tested negative or positive*** *for chlamydia. Check with the participant that she has received her result and that she is aware of it.]*

**Result: 🖵 negative 🖵 positive (tick the one that applies)**

**Q. 15 Could you tell me your experience in waiting for and receiving the test results?**

If negative now go to Question 16

If positive now go to Question 17

**Negative**

**16. If your test had shown you had chlamydia, how would you feel telling your partner about the result?**

| 1 | 2 | 3 | 4 | 5 |
| --- | --- | --- | --- | --- |
| Very uncomfortable | Uncomfortable | Neither comfortable or uncomfortable | Comfortable | Very comfortable |

[Explain that (a) notification of partners is usually discussed with women who are diagnosed with chlamydia (b) most men with chlamydia don’t have symptoms or signs of it and don’t know they are infected and (c) if sexual partners are treated this prevents re-infection of the woman.]

Go to last Q19.

**Positive**

**17. When you received your test results did you tell your partner?**

**Why/Why not?**

[Explain that (a) notification of partners is usually discussed with women who are diagnosed with chlamydia (b) most men with chlamydia don’t have symptoms or signs of it and don’t know they are infected and (c) if sexual partners are treated this prevents re-infection of the woman.]

**18. Can you tell us about your treatment experience?**

[Probe: further STI testing?]

TREATMENT – All participants regardless of + or -

**19. How would you feel/How did you feel about being treated for chlamydia whilst pregnant?**

**20. What do you think would be the best way to inform a sexual partner that they should get screened for Chlamydia?**

[Probe for the following: in person, using a contact letter, access to websites, text message, written information about chlamydia.]

**21. Do you have any other thoughts you would like to share that can help us decide the best way of offering pregnant women a test for chlamydia?**

Thank you for your time
